# Copy Number Variants of Uncertain Significance by Chromosome Microarray Analysis from Consecutive Pediatric Patients: Reevaluation Following Current Guidelines and Reanalysis by Genome Sequencing

**DOI:** 10.3390/genes16080874

**Published:** 2025-07-24

**Authors:** Wenjiao Li, Xiaolei Xie, Hongyan Chai, Autumn DiAdamo, Emily Bistline, Peining Li, Yuan Dai, James Knight, Abraham Joseph Avni-Singer, Joanne Burger, Laura Ment, Michele Spencer-Manzon, Hui Zhang, Jiadi Wen

**Affiliations:** 1 Department of Genetics, Yale University School of Medicine, New Haven, CT 06510, USA; wenjiao.li@yale.edu (W.L.); yutian_1029@163.com (X.X.); hongyan.chai@yale.edu (H.C.); autumn.diadamo@yale.edu (A.D.); emily.bistline@yale.edu (E.B.); peining.li@yale.edu (P.L.); j.knight@yale.edu (J.K.); michele.spencer-manzon@yale.edu (M.S.-M.); hui.zhang@yale.edu (H.Z.); 2 Department of Laboratory Medicine, Affiliated Qingyuan Hospital, Guangzhou Medical University (Qingyuan People’s Hospital), Qingyuan 511518, China; 3 School of Computing and Information, University of Pittsburgh, Pittsburgh, PA 15213, USA; yud108@pitt.edu; 4 Yale Center for Genome Analysis, Yale University, New Haven, CT 06516, USA; 5 Department of Pediatrics, Yale University School of Medicine, New Haven, CT 06510, USA; abraham.avni-singer@yale.edu (A.J.A.-S.); joanne.burger@yale.edu (J.B.); laura.ment@yale.edu (L.M.); 6 Department of Neurology, Yale University School of Medicine, New Haven, CT 06510, USA

**Keywords:** chromosomal microarray analysis (CMA), copy number variants of uncertain significance (CNVus), reevaluation, reclassification, reanalysis, whole genome sequencing (WGS)

## Abstract

Background: Copy number variants of uncertain significance (CNVus) from chromosome microarray analysis (CMA) presents unresolved challenges for clinical geneticists, genetic counselors, and patients. We performed a systematic reevaluation of reported CNVus and reanalysis of selected CNVus by whole genome sequencing (WGS) to assess the diagnostic value and clinical impact on CNVus reclassification. Methods: We retrospectively reviewed 5277 consecutive pediatric cases by CMA from the Yale Clinical Cytogenetics Laboratory over a 13-year period. Reevaluation was performed on all reported CNVus following current ACMG/ClinGen guidelines. Reanalysis by WGS was applied to selected cases for reclassification of CNVus. Results: A total of 567 CNVus from 480 cases were reported, which accounted for 9.1% of pediatric cases. A total of 4 CNVus in 4 cases (0.8%, 4/480) were reclassified to pathogenic/likely pathogenic CNVs (pCNVs/lpCNVs); while 23 CNVus in 23 cases (4.8%, 23/480) were reclassified to benign/likely benign CNVs (bCNVs/lbCNVs). The overall rate of reclassification was 5.6%. WGS performed on selected cases further defined breakpoints and ruled out additional causative genetic variants. Conclusions: The results from this study demonstrated the diagnostic value of periodic reevaluation of CNVus and reanalysis by WGS in an interval of 3–5 years and provided evidence to support standardized laboratory reevaluation and reanalysis.

## 1. Introduction

Chromosomal microarray analysis (CMA) has been used as the first-tier clinical diagnostic test for individuals with developmental disabilities (DD), intellectual disability (ID), autism spectrum disorder (ASD), or multiple congenital anomalies (MCAs) [1]. With its higher sensitivity in detecting submicroscopic copy number variants (CNVs) across the entire genome, CMA has shown significantly improved diagnostic accuracy and efficacy in comparison with conventional cytogenetic analysis [1,2,3]. However, CNV interpretation has been hindered due to the limited knowledge of gene-disease associations, especially on genes with dosage effects of haploinsufficiency and triple-sensitivity, incomplete penetrance, and variable expressivity, which caused inconsistencies in CNV classifications across laboratories, particularly prior to the implementation of the current American College of Medical Genetics and Genomics (ACMG) guidelines. Many CNVs reported as copy number variants of uncertain significance (CNVus) could complicate clinical decisions and raise patient anxiety [4]. To address this, the ACMG guidelines published in 2011 have been updated in 2020 with the introduction of a ClinGen semi-quantitative point-based scoring system to further standardize CNV interpretation [5,6]. Over the past decade, the expansion of publicly available databases (such as DGV Gold, Decipher, ClinGen, and ClinVar), advancements in computational pathogenicity prediction algorithms, and a growing body of functional studies have also facilitated the interpretation of CNVs.

The ACMG issued a statement in 2019 recommending that clinical laboratories periodically reevaluate and reanalyze genomic test results, including CMA, to ensure accurate and consistent variant classification [7]. However, only a few studies have assessed the clinical impact of systematic CMA reevaluation under the 2020 ACMG/ClinGen guidelines [8,9,10]. In this study, we retrospectively reviewed 567 CNVus from 5277 consecutive pediatric cases over a 13-year period at the Yale Clinical Cytogenetics Laboratory. The aims of this study were to assess the diagnostic value by reevaluating CNVus detected by CMA, the role of WGS reanalysis in further defining CNVus, and the clinical impact of CNVus reclassification.

## 2. Materials and Methods

### 2.1. Retrospective Review of Pediatric Cases

**Figure 1:**
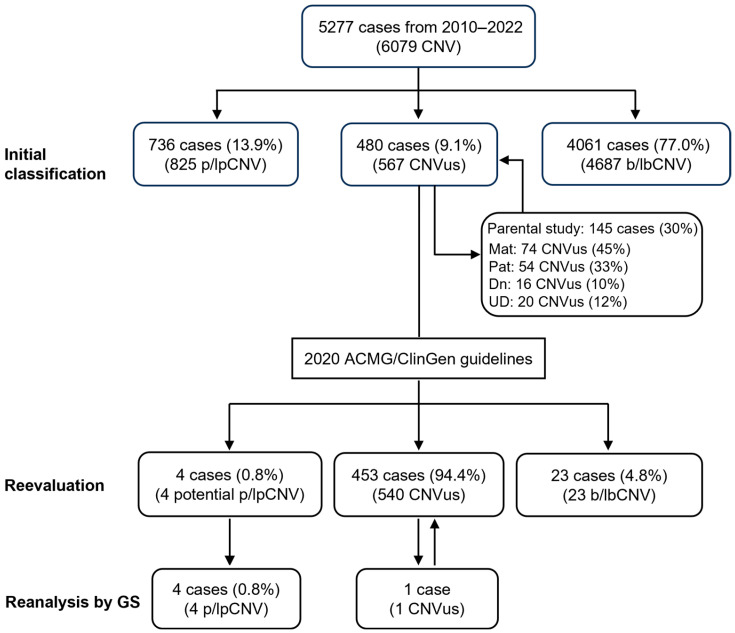
**Workflow for copy number variants of uncertain significance (CNVus) reevaluation and reanalysis.** A total of 5277 pediatric patients underwent chromosomal microarray analysis (CMA) from 2010 to 2022. Among them, 736 patients were identified with pathogenic or likely pathogenic CNV (p/lpCNV), 480 patients had CNVus, with 145 cases undergoing follow-up parental CMA study, and the remaining 4061 patients either had no CNV or only benign or likely benign CNV (b/lbCNV). The 567 CNVus from the 480 patients were reevaluated following the 2020 ACMG/ClinGen guidelines, resulting in a reduction in the number of CNVus through reclassification to either p/lpCNV or b/lbCNV. Five selected cases were further reanalyzed using whole genome sequencing (WGS). Mat: maternal; Pat: paternal; Dn: de novo; UD: undetermined.

A total of 5277 consecutive pediatric cases, aged from newborn to 17 years, were analyzed by CMA at the Yale Clinical Cytogenetics Laboratory over a 13-year period (2010–2022). From this case series, 567 CNVus were reported in 480 cases, and the results from the follow-up parental analyses were retrieved for this retrospective study. The workflow for reevaluation and reanalysis is shown in Figure 1. This retrospective chart review study was deemed exempt from institutional review board (IRB) and granted a waiver of consent based on the policy of the Yale University IRB.

### 2.2. DNA Extraction and Chromosomal Microarray Analysis

DNA extraction and CMA were performed following standardized procedures adopted from the manufacturers’ instructions. Genomic DNA was extracted from peripheral blood specimens using the Gentra Puregene Kit (Qiagen, Valencia, Santa Clarita, CA, USA). DNA quality and quantity were assessed using a NanoDrop spectrophotometer (Thermo Fisher Inc., Waltham, MA, USA) and further confirmed by agarose gel electrophoresis. For each sample, 1.5–2 µg of genomic DNA was used for CMA testing. The CMA analyses using Agilent’s SurePrint G3 Human Genome CGH  +  SNP microarray 180 K kit or 400 K kit (Agilent Technologies, Inc., Santa Clara, CA, USA) were performed as previously described [2,3]. Data were processed through the Agilent CytoGenomics Software 4.0 to generate a report of detected CNVs and regions of homozygosity with genomic and chromosomal visualization and genomic coordinates following the UCSC genome browser GRCh37/hg19 assembly (http://genome.ucsc.edu/). Deletions and duplications larger than 200 kilobases (Kb) are reported. However, smaller changes with pathogenic potential are also reported, while larger changes that are well-documented benign variants are not reported.

### 2.3. Reevaluation of CNVus

The 567 CNVus identified in 480 pediatric patients were manually curated by three geneticists independently, following the 2020 ACMG/ClinGen guidelines. The results were then discussed with the ordering healthcare provider. Briefly, gene content and CNV frequency in the general population were checked using UCSC (Feb. 2009 (GRCh37/hg19), tracks checked: Ensembl Genes, OMIM, Decipher, ClinGen CNVs, and DGV Structural Variants), DGV Gold (Build GRCh37: Feb. 2009, hg19), and gnomAD (v4.1.0). Dosage sensitivity scores for regions or genes were assessed using the ClinGen database (the version available as of November 2024; Tracks checked: Dosage Sensitivity and Gene-Disease Validity), while OMIM (the version available as of November 2024) was utilized to assess potential morbid genes within the regions. Additional assessments of CNV pathogenicity were performed using PubMed, DECIPHER (v11.31; tracks checked: Patient Variants, CNV Syndrome Variants, and Genes), and ClinVar (the version available as of November 2024).

### 2.4. Reanalysis by Whole Genome Sequencing (WGS)

Selected cases underwent WGS at the Yale Center for Genome Analysis (YCGA). In brief, archived DNA was sequenced on the Illumina platform (Illumina, Inc., San Diego, CA, USA). The YCGA pipeline integrates the commonly used Genome Analysis Toolkit 3 (GATK3) Best Practices workflows [11] and ANNOVAR (July, 2018) [12] for variant detection and annotation. CNVs were identified using a custom in-house algorithm. Briefly, only samples sequenced in the same flowcell are grouped for CNV detection to avoid potential batch effects. A YCGA-developed script automates the following: (1) computation of the average read depth for each sample in each exon; (2) computation of the mean depth for each exon; (3) computation of the normalized depths for each sample in each exon by dividing average read depth by whole exome average read depth; (4) computation of the mean of the normalized depths for each sample in each exon; (5) computation of the standard deviation (SD) of the normalized depth; (6) computation, for each sample in each exon, of each sample’s normalized depth over the mean of the normalized depths. If the normalized depth is less than/greater than 2 SD from the mean, the exon for the sample will be noted as a deletion or duplication, respectively. The mean sequencing coverage exceeded 50X, with over 96% of gene-containing regions (exons and intron-exon boundaries) covered at >50X. Genomic coordinates for copy number and sequence variants were designated according to the GRCh37/hg19 assembly in the UCSC Human Genome browser (http://genome.ucsc.edu/). The annotated data were analyzed for CNVs or sequence variants relevant to the patient’s phenotype. The exact positions of the breakpoints for the CNVus were examined using the bam file in the Integrative Genome Viewer (http://software.broadinstitute.org/software/igv/; accessed on 10 January 2023). Variants were filtered for relevance to human disease based on population frequency, information in disease-specific databases, and by in silico predictions of their impact on mRNA splicing or protein function.

## 3. Results

### 3.1. Characteristics of Pediatric Cases with Reported CNVus

From 2010 to 2022, CMA was performed on a total of 5277 pediatric cases at the Yale Clinical Cytogenetics Laboratory. Of these, 480 cases (9.1%) were reported to have at least one CNVus. These cases with CNVus were reevaluated, and their sex, age distribution, types of CNVus, and clinical indications were summarized in Table S1. Males significantly outnumbered females, with a ratio of 310 to 170. The patients’ ages ranged from the newborn period to 17 years (mean age: 4.2 ± 5.0 years), with 51% of patients aged less than 3 years. These 480 patients had a total of 567 CNVus identified, including 403 patients having one CNVus, 67 patients having two CNVus, and 10 patients having three CNVus. Among the 567 CNVus, 66% were duplications (*n* = 375), 30% were deletions (*n* = 172), and 3.5% were triplications or amplifications (*n* = 20). The most common indications for testing included developmental delay (*n* = 176, 36.7%), congenital anomalies (*n* = 96, 20%), autism spectrum disorders (*n* = 70, 14.9%), and seizures (*n* = 37, 7.7%). Parental studies were conducted for 145 patients (30%) with 164 CNVus (Figure 1). Of these, 74 CNVus (45%) were inherited from the mother and 54 (33%) from the father, with a total of 78% of CNVus being inherited; sixteen CNVus (10%) were defined as apparently *de novo* based on normal parental results. Furthermore, 20 CNVus (12%) remained undetermined as only one parent was tested.

**Figure 2:**
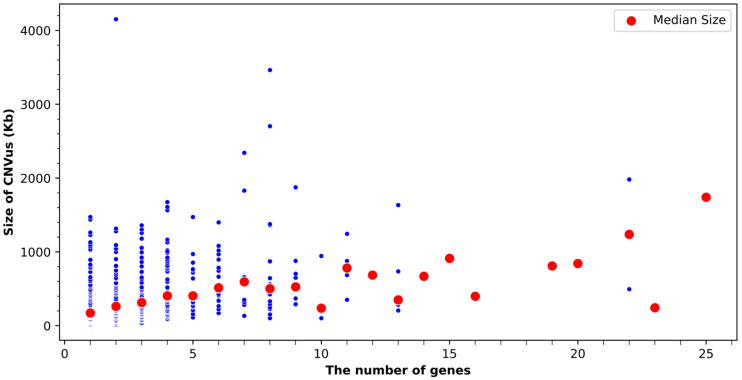
**Scatter plot showing the distribution of gene counts within each CNVus and their corresponding sizes.** Each blue dot represents an individual CNVus, while red dots represent the median CNVus size for each gene count category. There are no CNVus containing 17, 18, 21, or 24 genes. Kb: kilobase.

Most of the CNVus identified in this study were small and involved a limited number of genes (Figure 2). The sizes of these CNVus ranged from 9.8 Kb to 4152 Kb; with 79% being smaller than 600 Kb and 92% being smaller than 1000 Kb (Figure S1A). Additionally, 85% of the CNVus involved one to five genes, with 30% involving only a single gene (Figure S1B).

**Figure 3:**
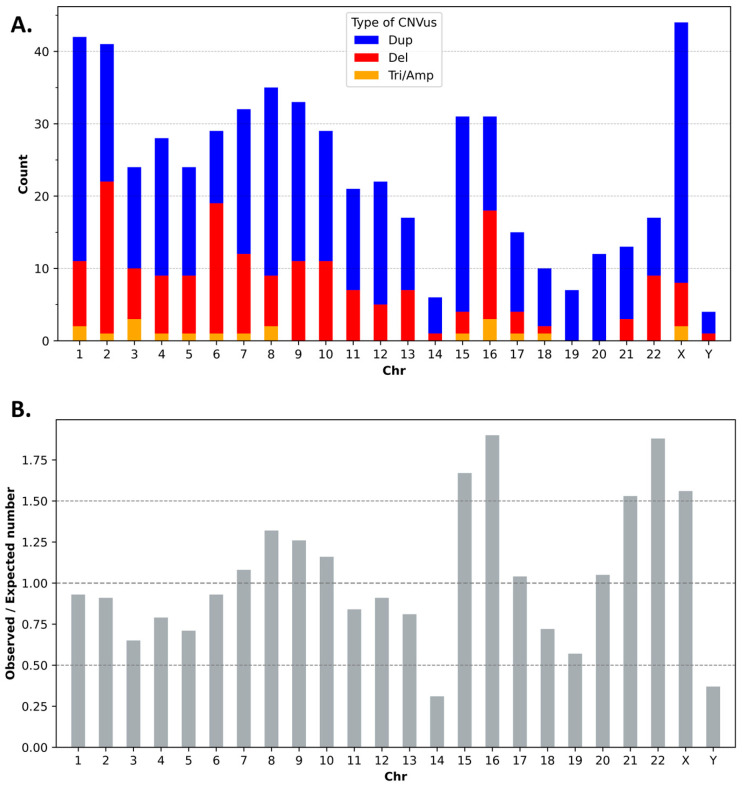
**Genomic distribution of reevaluated CNVus.** (**A**) Distribution of different types of CNVus per chromosome. Dup: duplication; Del: deletion; Tri/Amp: Triplication/Amplification; Chr: chromosome. (**B**) Number of CNVus per chromosome compared to the expected number, showing a non-random distribution pattern.

The 567 CNVus showed a non-random distribution across the genome compared to the expected distribution based on each chromosome’s proportional size relative to the entire genome (Figure 3, Table S2). Chromosomes 15, 16, 21, 22, and X harbored over 1.5 times the expected CNVus, while chromosomes 14 and Y had fewer than 0.5 times the expected number (Figure 3B, Table S2), aligning with the known uneven CNV distribution across the genome [13]. The relative abundance of low copy repeats (LCRs) on chromosomes 15, 16, 21, 22, and X likely explains their high CNV counts, while chromosome 14’s low CNV count could be due to the scarcity of LCRs and no well-characterized CNV hotspots [14,15].

### 3.2. Reclassified CNVus Follows Current ACMG/ClinGen Guidelines

**Figure 4:**
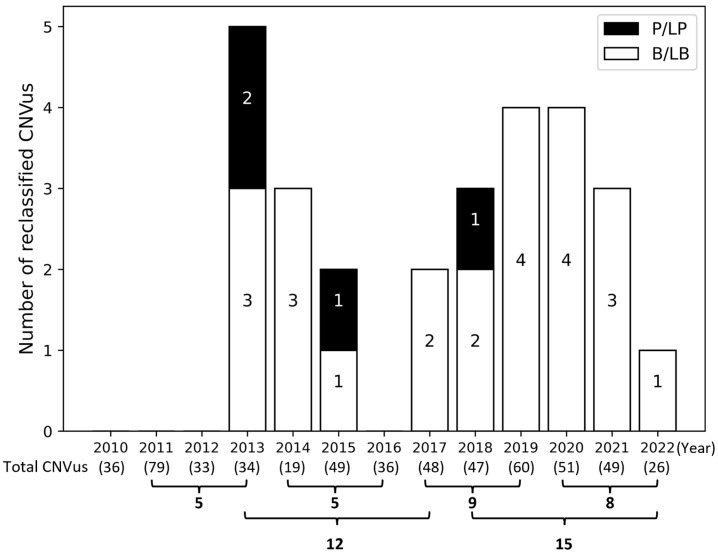
**Annual distribution of reclassified CNVus.** P/LP, pathogenic/likely pathogenic; B/LB, benign/likely benign. The total number of CNVus analyzed each year is indicated in parentheses below the corresponding year.

After reevaluating all 567 CNVus in 480 patients following the current ACMG/ClinGen guidelines, 27 CNVus in 27 patients (5.6%; 27/480) were reclassified. Among these, four CNVus in four patients were reclassified as pathogenic (p) or likely pathogenic (lp), primarily due to updates in public resources such as ClinGen Dosage Sensitivity, new evidence in the literature, and updated ACMG guidelines. This reclassification added a 0.8% (4/480) diagnostic yield. Additionally, 23 CNVus in 23 patients were reclassified as benign (b) or likely benign (lb), mainly due to updated population data in DGV Gold and gnomAD databases. All CNVus reclassified to pCNV/lpCNV were deletions, while the CNVus reclassified to bCNV/lbCNV were all duplications (Table 1). The reclassification rate showed some variation of 5, 5, 9, and 8 cases by a three-year interval but 12 and 15 cases by a five-year interval, respectively (Figure 4).

Two intragenic deletions in the *NRXN1*gene at 2p16.3 were reclassified. The first deletion was identified in a boy with mild ID, autism, seizures, and short stature (Table 1, case 1). This deletion was inherited from his mother, who has a history of seizures and depression. The second deletion was found in a boy with autism (Table 1, case 2). The *NRXN1* gene is linked to autosomal recessive Pitt–Hopkins-like syndrome 2 (OMIM #600565). However, in 2019 and 2021, ClinGen curated this gene, providing sufficient evidence for its association with an autosomal dominant complex neurodevelopmental disorder, with a HI score of 3 (https://search.clinicalgenome.org/kb/gene-dosage/HGNC:8008 accessed on 10 January 2023). *NRXN1* encodes the cell-surface receptor Neurexin-1-alpha, which binds neuroligins to form a calcium-dependent neurexin/neuroligin complex at central nervous system (CNS) synapses, essential for efficient neurotransmission and synapse formation. The first deletion of exons 1–5, including the promoter region of the NRXN1 α isoform, is predicted to result in an absent or disrupted protein product. The second deletion was initially detected as an in-frame deletion of exons 4–5 by CMA and further defined by WGS as an in-frame deletion of exons 3–5 (Table 2, case 2). Exons 4–5 deletion was reported in several unrelated individuals with disease, in one patient with global DD in Decipher (ID: 501209) and in families showing deletions segregated with affected members [16,17]. Based on the above evidence, these two deletions were reclassified as pCNV and lpCNV, respectively.

A 686.1 Kb deletion at 22q11.21 was identified in a boy presenting with DD and dysmorphic features (Table 1, case 3). This deletion was reclassified as an lpCNV. This deletion overlaps with the 22q11.2 recurrent region (central, B/C–D) (includes the *CRKL* gene), which currently has a ClinGen HI score of 2. Literatures provided emerging evidence that deletions of this region are associated with a variable clinical phenotype that may include DD, dysmorphic facial features, CNS anomalies, ID, behavioral problems, skeletal anomalies, cardiovascular defects, genitourinary anomalies, and immune deficiency [18,19,20,21,22]. Additionally, this region contains the *CRKL* gene, which has a ClinGen HI score of 1. Mouse models indicated that haploinsufficiency of *CRKL* may contribute to the 22q11.2 phenotype, particularly regarding cardiovascular and genitourinary defects [22,23].

A 188.9 Kb deletion at Xp21.1 was reclassified as a pCNV in a girl presenting with global DD, hearing loss, and MCAs (Table 1, case 4). This reclassification was based on updated ACMG guidelines for interpreting losses involving X-linked recessive genes in females, which recommend assessing classification based on the predicted impact in males, even though the overall clinical significance may be uncoupled. This deletion involves exons 49–53 of the *DMD* gene, which is predicted to result in an in-frame deletion. The exons 49–53 deletion has been previously reported in a male with a clinical diagnosis of Becker muscular dystrophy (OMIM#300376) [24]. Additionally, smaller in-frame deletions, such as those involving exon 49 [25,26], exons 49–51 [27], or exons 51–52 [28], have also been reported in affected male patients. This finding likely represents carrier status. However, unfavorably skewed X-inactivation could result in clinical features related to this disorder, such as DD and hearing loss, though it does not account for the MCAs. The patient underwent exome sequencing (ES) at another laboratory, which identified two heterozygous variants of uncertain significance (VUSs) in the *EFTUD2* and *GLI2* genes, potentially contributing to the MCAs.

Twenty-three CNVus in three groups were reclassified to lbCNV/bCNV (Table 1, groups 1–3), primarily based on updated population data from the DGV Gold and gnomAD databases. The 23 reclassified CNVus were all duplications. The duplications at 9p24.3 (Group 1) and at 15q13.3 (Group 3) were reclassified as lbCNV due to the presence of multiple similar duplications in the general population, their absence in disease populations, and the lack of dosage-sensitive regions or genes according to the ClinGen database. The 15q11.1q11.2 duplication (Group 2) was reclassified as bCNV due to its overlap with a common population CNV based on data from DGV Gold and gnomAD.

Lastly, four CNVus remained current classification but were monitored for emerging evidence of pathogenicity (Table S3). The first CNVus is a 285.1 Kb deletion at 1q43q44 (Table S3, case 1). This deletion overlaps with the recurrent 2 Mb 1q43q44 terminal deletion regions (includes the *AKT3* gene, which is currently an established dosage sensitive region with a haploinsufficiency (HI) score of 3 by ClinGen (https://search.clinicalgenome.org/kb/gene-dosage/region/ISCA-37493 accessed on 10 January 2023). Although the detected CNV is significantly smaller than the recurrent 2 Mb deletion, the minimum critical region has not been clearly defined. The *AKT3* gene within this CNVus was assigned a haploinsufficiency score of 1 by ClinGen in 2021, which could be considered a potential candidate gene for brain anomalies [29]. Similarly, three intragenic deletions in the *CNTNAP2* gene were noticed (Table S3, cases 2–4). *CNTNAP2* was curated by ClinGen in 2019 (HI score of 1), highlighting its emerging association with autosomal dominant complex neurodevelopmental disorders, including ID, DD, epilepsy, schizophrenia, Tourette syndrome, and increased susceptibility to autism [30,31,32,33]. While the pathogenic role of heterozygous *CNTNAP2* variants remains controversial, a few potential causative variants have been reported in patients since the 2019 ClinGen curation [34,35]. For these four CNVus, the current data remained insufficient to confirm their causative role in the disease, but monitoring for emerging evidence of pathogenicity for reclassification was warranted.

### 3.3. Reanalysis by Genomic Sequencing (WGS)

**Figure 5:**
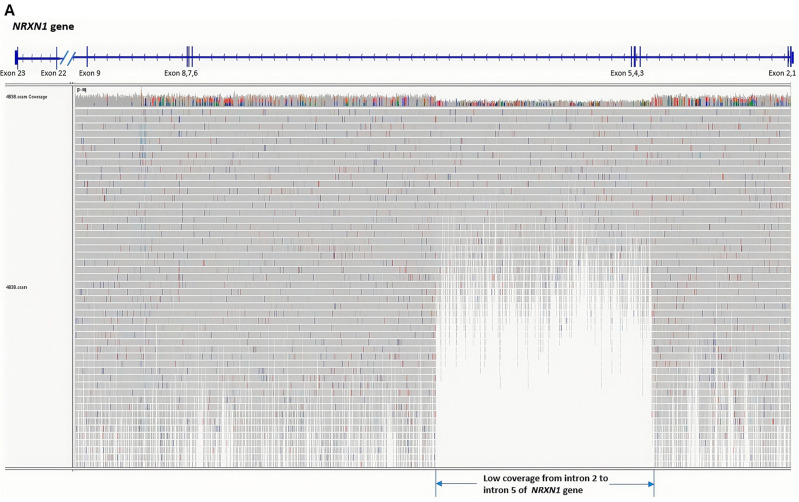

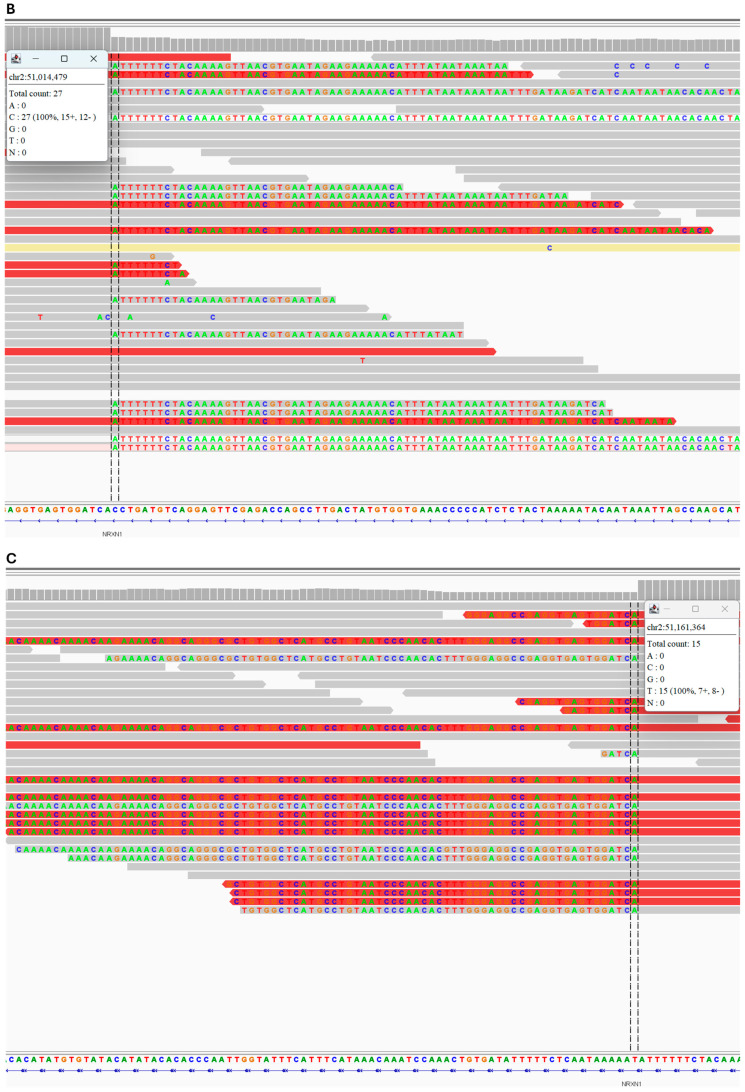
**Whole genome sequencing analysis of Case 2.** (**A**) Integrative Genomics Viewer (IGV) visualization of NRXN1 gene coverage, highlighting a low coverage from intron 2 to intron 5. (**B**) IGV view of the left breakpoint with the corresponding junction fragments shown in the rainbow sequence. (**C**) IGV view of the right breakpoint with the corresponding junction fragments shown in the rainbow sequence.

The current ACMG guidelines highlight the importance of reading frame determination of intragenic CNVs, particularly those with known clinical implications, such as CNVs in the *DMD* gene. In this study, three out of four CNVs reclassified to pCNV/lpCNV are intragenic, including one in the *DMD* gene. This prompted us to perform WGS to precisely delineate the breakpoints. Five cases were reanalyzed by WGS, and the breakage-fusion sequences of these CNVus defined by WGS and their functional implications are summarized in Table 2. In the two cases with an intragenic CNVus in the *NRXN1* gene (Table 2, cases 1 and 2), WGS confirmed the deletions detected by CMA and further delineated frameshift exon deletion or in-frame exon deletion for their reclassification. In particular, case 2 was found to have a deletion affecting exons 3–5, rather than exons 4–5 as originally indicated by CMA (Figure 5). The CNVus of a deletion at 22q11.21 (Table 2, case 3) was confirmed by WGS and indicative of a deletion mediated by LCR-B and LCR-D at 22q. In case 4, WGS confirmed the in-frame deletion of exons 49–53 in the *DMD* gene. Accurate reading frame determination is particularly crucial due to its clinical significance in distinguishing between Duchenne and Becker muscular dystrophy [36]. One patient (Table 2, case 5) with an intragenic CNVus in the *NRXN1* gene (HI score of 3) was reanalyzed by WGS to delineate the exact breakage-fusion sequence and confirmed that the deletion was restricted to intron 5 with no involvement of the adjacent exons. WGS confirmed all CNVus detected by CMA, delineated nucleotide-level breakage-fusion sequences to assess their impact on gene transcription for interpretation on reclassification, and also ruled out other clinically significant CNVs or sequence variants undetected by CMA.

## 4. Discussion

The reporting rate for CNVus in our laboratory is 9.1%, which is comparable with ranges reported in the literature [1,37,38]. The majority (66%) of reported CNVus are duplications. Most CNVus (92%) are smaller than 1000 Kb, and 85% of CNVus involve only one to five genes. This distribution aligns with the observation that duplications and small CNVs are more often classified as CNVus, due to incomplete reference of their occurrence in the general population, unclear gene–disease association, and limited studies on dosage effect by haploinsufficiency and triplosensitivity [6].

Our study demonstrates that periodic reevaluation of CNVus can have a significant clinical impact. In our case series, the reclassification rate was 5.6%, with 0.8% of CNVus reclassified to pCNV/lpCNV, thus providing a diagnosis for four patients. Recontacting patients with reanalyzed and reclassified results could enable more tailored clinical care for them and their families by: (1) reducing unnecessary additional testing by providing a definitive diagnosis; (2) establishing a basis for appropriate genetic counseling, such as help families assess genetic risks for future offspring, especially as pediatric patients approach reproductive age and consider family planning; and (3) guiding clinical management for related health issues and proper interventions. Additionally, 23 patients (4.8%) had CNVus reclassified as bCNV/lbCNV, which alleviated unnecessary anxiety and helped prevent unwarranted clinical interventions and family planning concerns, although testing may still be required to identify other possible genetic or non-genetic contributors to the patients’ conditions.

Although reanalysis of WGS or ES data every 1–2 years is widely recommended and has become standard practice in many clinical laboratories [39,40], there are no clear recommendations or consensus regarding the optimal interval for CNVus reevaluation, and no studies have specifically addressed this question. Our data suggests that reevaluating CNVus within a 3–5 year period is a reasonable and feasible approach. While periodic CNVus reevaluation is important, it requires significant time and effort from both laboratories and clinicians. Therefore, it is crucial to develop standardized procedures that maximize clinical impact while minimizing the burden on laboratories and healthcare systems.

This study also highlights the importance of follow-up parental testing for evaluating the pathogenicity of CNVus. Parental testing helps clarify a CNVus’s clinical relevance by identifying whether it is inherited or *de novo. De novo* CNVus, which occur spontaneously, are more likely to be disease-associated, whereas inherited CNVus are often benign, especially if present in an unaffected parent. In this study, an inherited CNVus observed in a parent with potentially related, though non-specific, phenotypes (Table 1, case 1) supported its reclassification as pathogenic. Additionally, incomplete penetrance and variable expressivity were noted for this CNV. The presence of common, non-specific phenotypes in parents, along with incomplete penetrance and variable expressivity, complicates CNV classification. Non-specific phenotypes may arise from diverse genetic and environmental factors and are not necessarily related to the CNV in question. Incomplete penetrance and variable expressivity add further complexity, as a pCNV may not always manifest in carriers or may present differently across individuals. Despite these challenges, parental testing remains a valuable approach in CNV interpretation, as identifying *de novo* variants or tracking inheritance patterns provides critical insights into pathogenicity.

This study underscores the crucial role of WGS-based reanalysis, especially for intragenic CNVus. Current ACMG/ClinGen guidelines also recommend this approach [6]. WGS not only delineated breakage–fusion sequences but also determined if the reading frame is disrupted, enabling better predictions of their impact on gene function and interpretation. WGS is also essential for ruling out other causative CNV or sequence variants undetected by CMA, providing additional evidence to enhance reclassification. However, interpreting intragenic in-frame or intronic deletions and duplications confirmed by WGS remains challenging, especially when functional studies are limited, and no similar CNVs are reported in medical literature or databases. Integrating bioinformatic predictions or functional studies on specific genes in the affected organ or system could reveal underlying dosage-sensitive mechanisms and improve CNVus reclassification [41].

The reported CNVus reclassification rate varies greatly among published studies. Jean-Marie et al. (2023) conducted a retrospective review of 1641 CMA cases over an 8-year period (2010–2017) and found a significantly higher reclassification rate of 40.9% (106/259) for CNVus, with the majority (89%; 94/106) reclassified to b/lb and 11.3% (12/106) reclassified to p/lp [8]. Similarly, Midhat et al. (2020) reported an even higher reclassification rate of 75% (163/216) for CNVus from 2011 to 2013, where 96% were reclassified to b/lb and only 4% reclassified to p/lp [9]. In contrast, George et al. (2024) reported a much lower reclassification rate of 0.7% (15/2137) in a review of 16,000 CMA postnatal cases over seven years (2010–2017) [10]. This variation in reclassification rates is likely attributable to inconsistent classification and reporting criteria for CNVs across different laboratories prior to the publication of the 2020 ACMG/ClinGen guidelines, as well as differences in sample size and study duration. Our study, along with other published findings, emphasizes the need for standardized CNV classification and highlights the pivotal role of ClinGen dosage pathogenicity curations. The 2020 ACMG/ClinGen CNV classification guidelines introduced a more rigorous, evidence-based approach aimed at reducing inter-laboratory discrepancies, offering a valuable framework for CNV reevaluation, especially for CNVs reported prior to 2020. Strict adherence to current ACMG guidelines is crucial for minimizing discrepancies and ensuring accurate variant interpretation. CNVus reevaluation is a unique aspect of genomic medicine, reflecting a continually evolving body of research and literature. Our study, along with others, provides evidence and reinforces the importance of periodic reevaluation of CNVus as genetic knowledge advances and ACMG guidelines evolve. Furthermore, timely communication with referring physicians with updated reports for patient re-contact is important. The latest ACMG’s policy underscores that re-contact is a shared responsibility among healthcare providers, laboratories, and patients. Recommendations from the European Society of Human Genetics also highlight the need for collaborative effort [42,43]. Therefore, more studies with multidisciplinary input from diverse professional groups are needed for developing comprehensive guidelines on CNVus reevaluation, reanalysis, amended reporting, and patient re-contact.

## 5. Conclusions

The results from this study demonstrated the diagnostic value and clinical significance of periodic CNVus reevaluation and WGS-based reanalysis. WGS should be implemented to accurately delineate breakpoints, particularly for intragenic CNVus, and rule out other causative genetic variants undetected by CMA. Our data suggested that a standardized procedure of reevaluation and reanalysis every 3–5 years, along with the updated clinical database, is practical. Additionally, parental testing provided valuable evidence for CNVus reclassification, enabling more precise genetic counseling for families. This study also highlighted the importance of timely communication with referring physicians for updated reporting and patient re-contact after reclassification, ultimately improving patient care.

## Figures and Tables

**Table 1:** Reclassified CNVus.

Case # (Report Date)	CMA *^a^* (hg19)	Size (Kb)	Gene	New Class	Semiquantitative Score Per ACMG Technical Standards	Overlap with Dosage Sensitive Gene/Region	Recurrent	Case Number	Indication	Sex	Age Range (Year)	Explain Patient Phenotypes	Other Genetic Testing
1 (2013)	2p16.3 del (mat) *^b^*	178.9	NRXN1: estimated exons 1–5 del	P *^e^*	1	NRXN1 gene (HI score 3)	Yes	1	mild ID *^i^*, autism, seizures, short stature	M	3–6	Yes	Normal karyotyping analysis; Negative Fragile X testing
2 (2015)	2p16.3 del *^c^*	129	NRXN1: estimated in-frame exons 4–5 del	LP *^f^*	0.9	NRXN1 gene (HI score 3)	Yes	1	Autism	M	3–6	Yes	N/A *^m^*
3 (2013)	22q11.21 del	686.1	ZNF74, KLHL22, MED15, POM121L4P, PI4KA, SERPIND1, SNAP29, CRKL, AIFM3, LZTR1, P2RX6, SLC7A4	LP	0.9	22q11.21 recurrent region (includes CRKL), region HI score:2; CRKL: HI score 1	Yes	1	DD *^j^*, dysmorphic features	M	9–12	Yes	Negative Fragile X testing
4 (2018)	Xp21.1 del (mat)	188.9	DMD: estimated in-frame exons 49–53 del	P	1	DMD gene (HI score 3)	No	1	GDD *^k^*, hearing loss, MCAs *^l^*	F	12–15	Partially; cannot explain MCAs	Exome Sequencing identified two heterozygous VUSs in *EFTUD2* and *GLI2* genes
Group 1 (2013–2022)	9p24.3 dup *^d^*	671.4	C9orf66, DOCK8, KANK1 and DMRT1	LB *^g^*	−0.9	No	Yes	7					
Group 2 (2017–2020)	15q11.1q11.2 dup	3157.6	GOLGA6L6, POTEB, OR4N4, GOLGA6L1, TUBGCP5, CYFIP1, NIPA2 and NIPA1	B *^h^*	−1	Overlaps with a common population variation with frequency at 14.36% in DGV gold	Yes	5					
Group 3 (2013–2021)	15q13.3 dup	489.1	CHRNA7	LB	−0.9	No	Yes	11					

*^a^* CMA, chromosomal microarray analysis; *^b^* mat, maternal; *^c^* del, deletion; *^d^* dup, duplication; *^e^* P, pathogenic; *^f^* LP, likely pathogenic; *^g^* LB, likely benign; *^h^* B, benign; *^i^* ID, intellectual disability; *^j^* DD, developmental delay; *^k^* GDD, global developmental delay; *^l^* MCAs, multiple congenital anomalies; *^m^* N/A, not available. #: indicates the case number assigned to each individual or group. Estimated breakpoints are defined based on the primary transcript of each gene. NRXN1, NM_001330078.2; DMD, NM_004006.3.

**Table 2:** Cases reanalyzed by whole genome sequencing (WGS).

		CMA *^a^* (hg19)		Size (bp)		Gene(s) Involved and Breakpoints (BP *^c^*)	
Case #	Chr		WGS *^b^* (hg19)	CMA	WGS	CMA	WGS
1	2p16.3	(51100412_51279305)x1 mat *^d^*	g.51095598_51283628del *^e^*	178,893	188,030	NRXN1 (frameshift exons 1–5 del) Left BP: upstream of 5’UTR Right BP: intron 5	NRXN1 (frameshift exons 1–5 del) Left BP: upstream of 5’UTR; Right BP: intron 5
2	2p16.3	(51021452_51149974)x1	g.51014479_51161364del	128,522	146,885	NRXN1 (in-frame exons 4–5 del) Left BP: intron 3 Right BP: intron 5	NRXN1 (in-frame exons 3–5 del) Left BP: intron 2 Right BP: intron 5
3	22q11.21	(20754422_21440514)x1	LCR *^f^*: B–D	686,092	LCR: B–D	ZNF74, KLHL22, MED15, POM121L4P, PI4KA, SERPIND1, SNAP29, CRKL, AIFM3, LZTR1, P2RX6, SLC7A4	ZNF74, KLHL22, MED15, POM121L4P, PI4KA, SERPIND1, SNAP29, CRKL, AIFM3, LZTR1, P2RX6, SLC7A4
4	Xp21.1	(31691769_31880635)x1 mat	g.31685100_31882273del	188,866	197,173	DMD (in-frame exons 49–53 del) Left BP: intron 48 Right BP: intron 53	DMD (in-frame exons 49–53 del) Left BP: intron 48 Right BP: intron 53
5	2p16.3	(50929220_50982172)x1	g.50928799_50986959del	52,952	58,160	NRXN1: intronic del in intron 5	NRXN1: intronic del in intron 5

*^a^* CMA, chromosomal microarray analysis; *^b^* WGS, whole genome sequencing; *^c^* BP, breakpoint; *^d^* mat, maternal; *^e^* del, deletion; *^f^* LCR, low copy repeat. #: indicates the case number assigned to each individual. Breakpoints are defined based on the primary transcript of each gene. NRXN1, NM_001330078.2; DMD, NM_004006.3.

## Data Availability

All data supporting the findings of this study are available within the paper and its Supplementary Materials. Raw data files regarding patient clinical data are not openly available due to reasons of sensitivity and study participant privacy; however, deidentified raw data are available from the corresponding author upon reasonable request.

## Supplementary Materials

The following supporting information can be downloaded at: https://www.mdpi.com/article/10.3390/genes16080874/s1. Figure S1: Size and gene content of reevaluated CNVus. (A) Size distribution of CNVus, with the percentage of cases for each size category indicated in the corresponding group’s bar. (B) Number of protein-coding RefSeq genes within the CNVus, with the percentage of cases for each gene count category indicated in the corresponding group’s bar. Table S1: Characteristics of the 480 pediatric cases with 567 reported CNVus. Table S2. Genomic distribution of reevaluated CNVus. Table S3. CNVus pending emerging evidence for pathogenicity.
