# Supplementary material for: Copy Number Variants of Uncertain Significance by Chromosome Microarray Analysis from Consecutive Pediatric Patients: Reevaluation Following Current Guidelines and Reanalysis by Genome Sequencing
Source: Genes (Basel). 2025 Jul 24;16(8):874. doi: 10.3390/genes16080874 (PMC12385847; doi:10.3390/genes16080874)
Supplement: Supplementary file 1 [file genes-16-00874-s001.zip › genes-3748566-supplementary.pdf]

Figure S1.

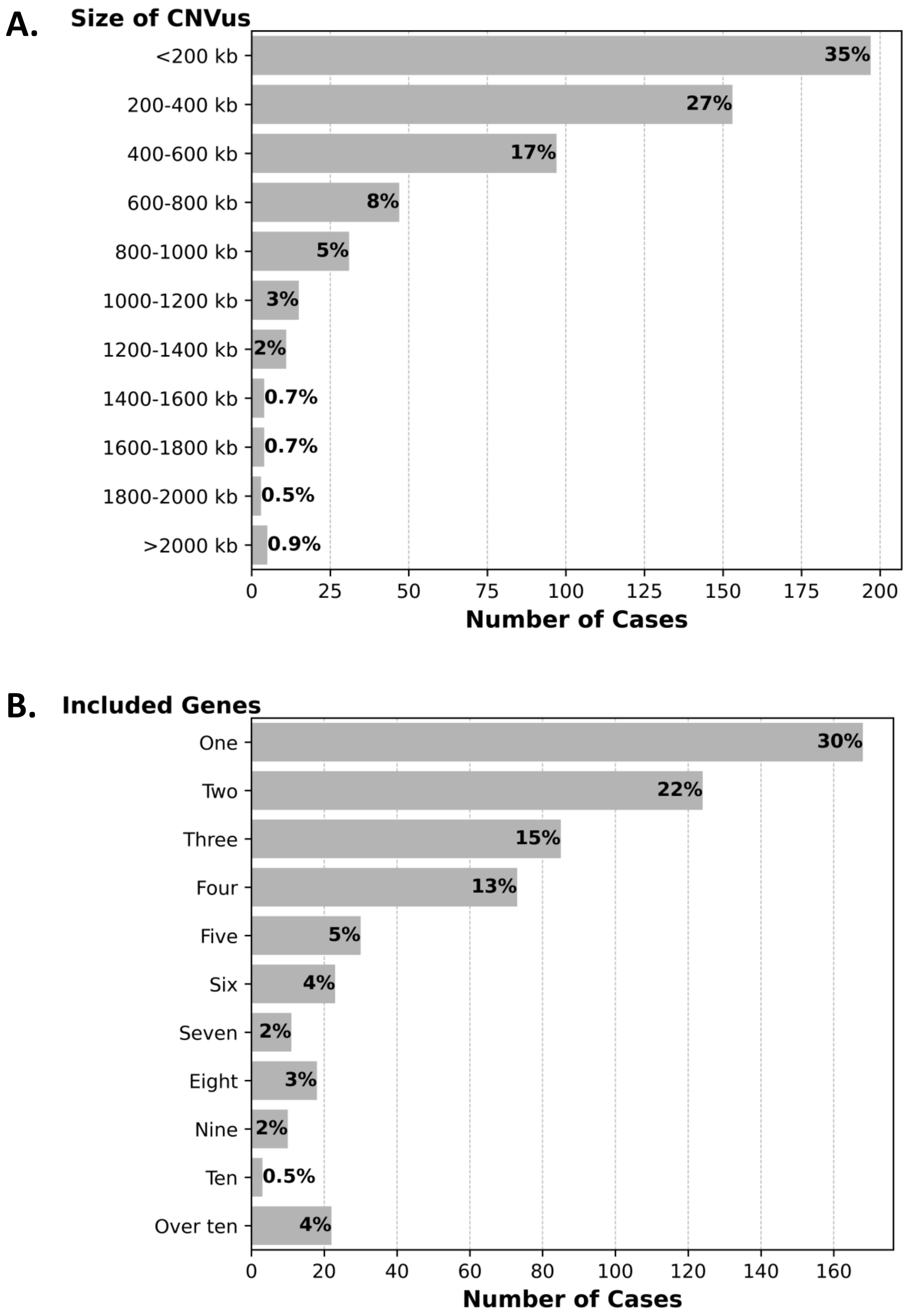

Figure S1. Size and gene content of reevaluated CNVus. (**A**) Size distribution of CNVus, with the percentage of cases for each size category indicated in the corresponding group's bar. (**B**) Number of protein-coding RefSeq genes within the CNVus, with the percentage of cases for each gene count category indicated in the corresponding group's bar.

Table S1. Characteristics of the 480 pediatric cases with 567 reported CNVus

**Number of patients (N=480)**	
Female	170 (35%)
Male	310 (65%)
Age (Mean ± sd)	4.2 ± 5.0
age <3	243 (51%)
3≤ age <6	85 (18%)
6≤ age <9	49 (10%)
9≤ age <12	39 (8%)
12≤ age <15	40 (8%)
15≤ age <18	24 (5%)
**Types of CNVus (N=567)**	
Deletion	172 (30%)
Duplication	375 (66%)
Triplication/amplification	20 (3.5%)
**Indication**	
Developmental delay ± other	176 (36.7%)
Congenital anomaly (isolated or multiple)	96 (20%)
Autism ± other	70 (14.9%)
Seizures ± other	37 (7.7%)
Intellectual disability ± other	13 (2.7%)
Other	148 (30.8%)

Table S2. Genomic distribution of reevaluated CNVus.

Total CNVus (n=567)	Chr1	Chr2	Chr3	Chr4	Chr5	Chr6	Chr7	Chr8	Chr9	Chr10	Chr11	Chr12	Chr13	Chr14	Chr15	Chr16	Chr17	Chr18	Chr19	Chr20	Chr21	Chr22	ChrX	ChrY
Tri/amp	2	1	3	1	1	1	1	2	0	0	0	0	0	0	1	3	1	1	0	0	0	0	2	0
Del	9	21	7	8	8	18	11	7	11	11	7	5	7	1	3	15	3	1	0	0	3	9	6	1
Dup	31	19	14	19	15	10	20	26	22	18	14	17	10	5	27	13	11	8	7	12	10	8	36	3
Observed number	42 (7.4%)	41 (7.2%)	24 (4.2%)	28 (4.9%)	24 (4.2%)	29 (5.1%)	32 (5.6%)	35 (6.2%)	33 (5.8%)	29 (5.1%)	21 (3.7%)	22 (3.9%)	17 (3%)	6 (1.1%)	31 (5.5%)	31 (5.5%)	15 (2.7%)	10 (1.8%)	7 (1.2%)	12 (2.1%)	13 (2.3%)	17 (3.0%)	44 (7.8%)	4 (0.7%)
Expected number[a]	46 (8.0%)	45 (7.9%)	37 (6.5%)	35 (6.2%)	33 (5.9%)	31 (5.5%)	29 (5.2%)	27 (4.7%)	26 (4.6%)	25 (4.4%)	25 (4.4%)	24 (4.3%)	21 (3.7%)	20 (3.5%)	18 (3.3%)	16 (2.9%)	14 (2.6%)	14 (2.5%)	12 (2.1%)	11 (2.0%)	9 (1.5%)	9 (1.6%)	29 (5.0%)	11 (1.9%)

[a]Expected CNVus number was calculated based on the proportional size of each chromosome to the total genome.

Table S3. CNVus pending emerging evidence for pathogenicity.

Case # (report date)	CMA[a] (hg19)	Size (Kb)	Gene	Overlap with dosage sensitive gene/region	Recurrent	case number	Indication	Gender	Age range (Year)	Explain patient phenotypes	Other genetic testing
1 (2012)	1q43q44 del[b]	285.1	SDCCAG8, AKT3	1q43q44 terminal region (includes AKT3), region HI score 3; AKT3: HI score 1	No	1	DD[c]	M	9-12	Yes	Normal karyotyping analysis
2 (2013)	7q35 del	270.5	CNTNAP2: estimated frameshift exons 2-3 del	CNTNAP2 gene (HI score 1); emerging evidence for its association with autosomal dominant complex neurodevelopmental disorders	Yes	1	Autism	M	0-3	Yes	N/A[d]
3 (2013)	7q35 del	241.4	CNTNAP2: estimated frameshift exons 2-3 del	Same as above	Yes	1	DD	M	0-3	Yes	N/A
4 (2018)	7q35 del	65.8	CNTNAP2: estimated in-frame exons 5-8 del	Same as above	Yes	1	Seizures, facial hemangioma, periventricular heterotopia	F	0-3	Partially; can't explain facial hemangioma, periventricular heterotopia	Normal karyotyping analysis

[a]CMA: chromosomal microarray analysis; [b]del: deletion; [c]DD: developmental delay; [d]N/A: not available. Estimated breakpoints are defined based on the primary transcript of the gene. CNTNAP2: NM_014141.6.
